# Supplementary material for: The structure of a tetrameric septin complex reveals a hydrophobic element essential for NC-interface integrity
Source: Commun Biol. 2024 Jan 6;7:48. doi: 10.1038/s42003-023-05734-w (PMC10771490; doi:10.1038/s42003-023-05734-w)
Supplement: Supplementary file 2 — Supplementary Information [file 42003_2023_5734_MOESM2_ESM.pdf]

Supplementary information

A

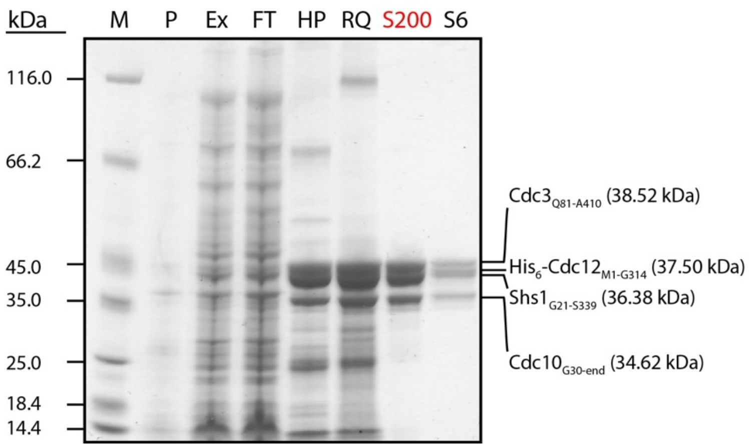

B

Septin	No. of peptides	Intensity
**Cdc10**	131	1.81E+10
**Cdc3**	140	1.61E+10
**Cdc12**	171	1.38E+10
**Shs1**	100	1.64E+10

Supplementary Figure 1. Characterization of the septin complex preparation for crystallography. **A.** Coomassie stained SDS-PAGE showing a purification setup. M-marker; P – pellet; Ex – soluble extract; FT – flowthrough from IMAC column; HP – pooled eluate from IMAC; RQ – pooled eluate from IEX; S200 – pooled product peak from preparative SEC used for crystallization, S6 – pooled product peak from analytical size exclusion. An unprocessed scan of the gel with original size marker is shown. **B.** Mass spectrometry-based analysis of the septin complex purification used for crystallization. The number of identified peptides and the peptide abundance is provided. The subunits are present in nearly stoichiometric order.

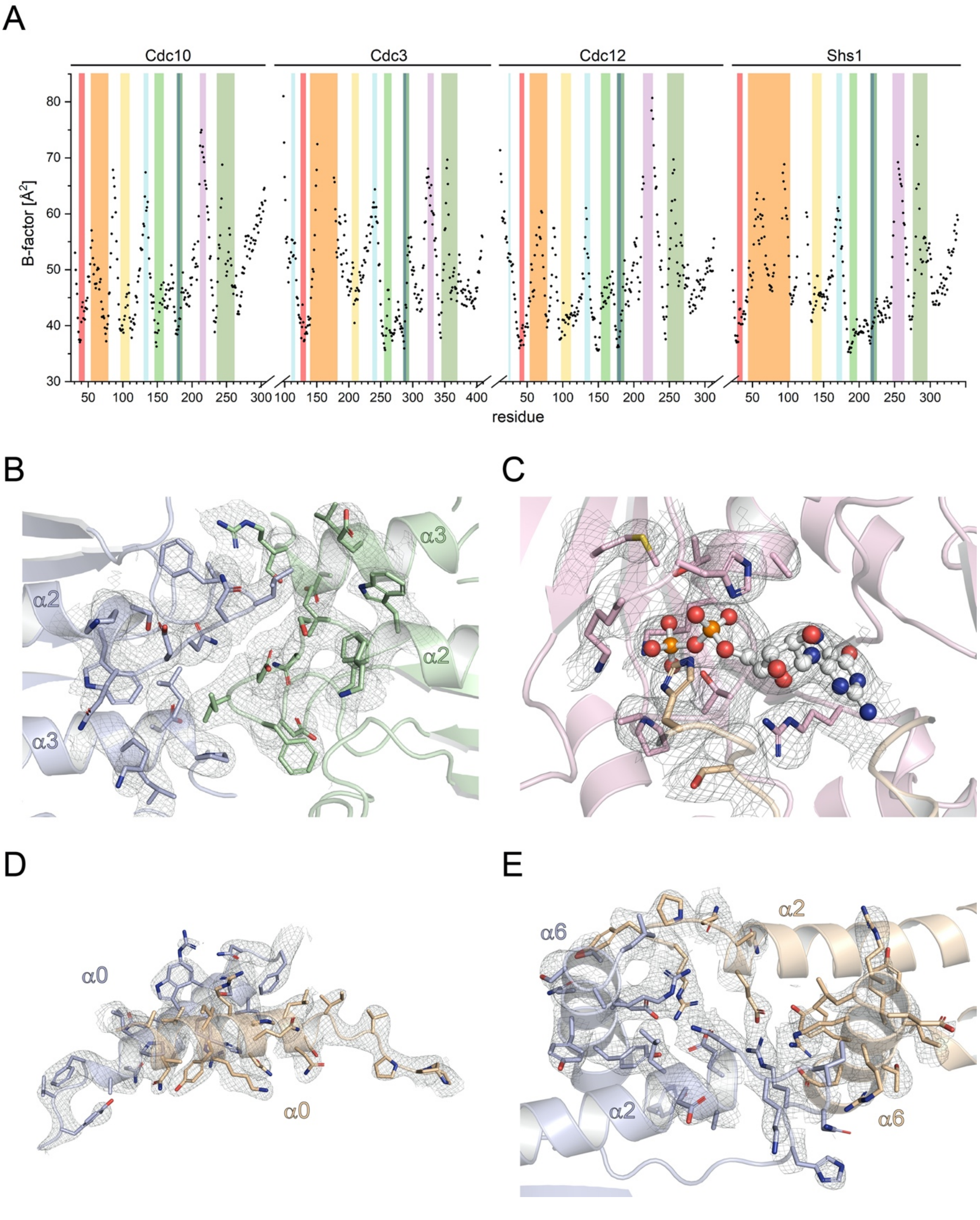
A
Cdc10
Cdc3
Cdc12
Shs1
B-factor [Å²]
residue
B
α2
α3
α3
α2
C
D
α0
α0
E
α6
α2
α2
α6

Supplementary Figure 2. Representative electron density display. **A.** B-factors of all Cα atoms in the structure. **B.** Representative 2mFo-DFc electron density map of the upper part of the Cdc3-Cdc10 G interface. **C.** Representative 2mFo-DFc electron density map of the 5 Å environment around the phosphate groups of the GDP in Shs1. **D.** Representative 2mFo-DFc electron density map of the α0-helices in the Cdc12-Cdc3 NC interface. **E.** Representative 2mFo-DFc electron density map of the upper part of the Cdc12-Cdc3 NC interface.

Electron density maps are contoured at a σ-level of 1.0. The colour code of the structural elements and subunits is as in Fig. 1 (Cdc3: light-blue, Cdc10: smudge, Cdc12: wheat, Shs1: light-pink).

Cdc10-Cdc10 (0.873)

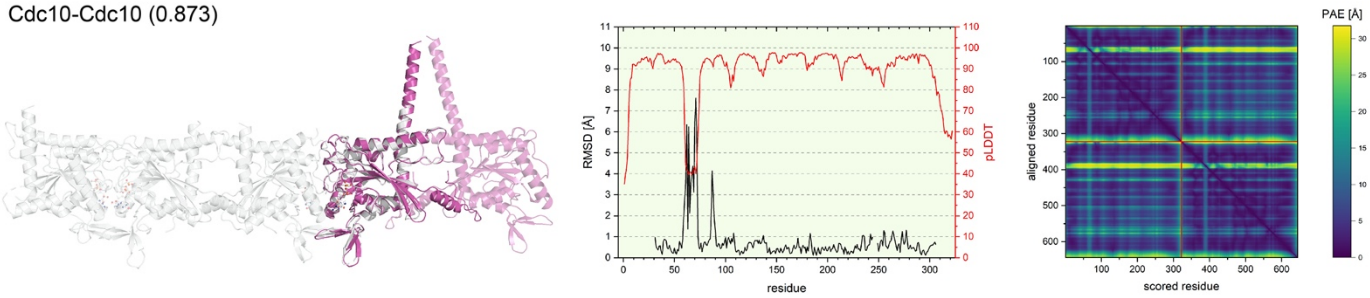

Cdc10-Cdc3$_{Q81\text{-}L412}$ (0.860)

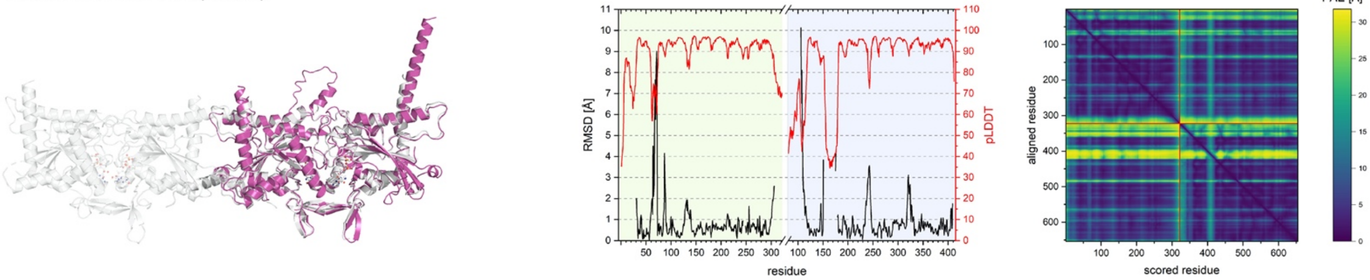

Cdc3$_{Q81\text{-}L412}$-Cdc12$_{M1\text{-}T317}$ (0.880)

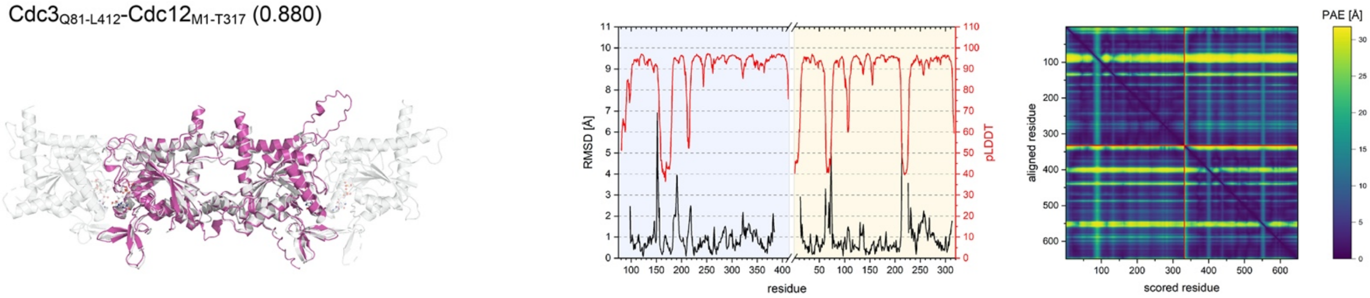

Cdc12$_{M1\text{-}T317}$-Shs1$_{M1\text{-}S339}$ (0.881)

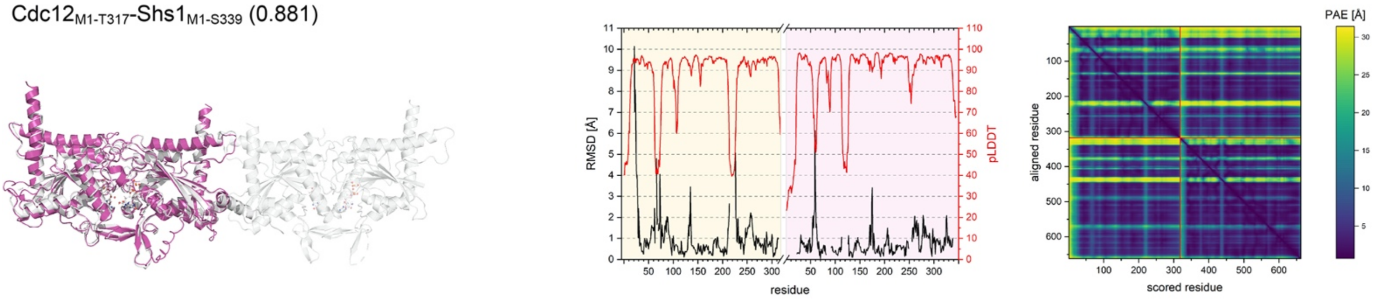

Cdc11-terminal tetramer (0.696)

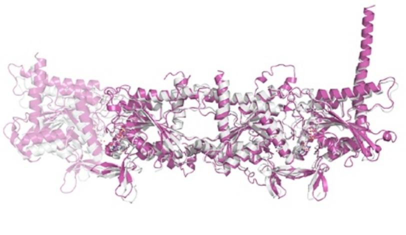

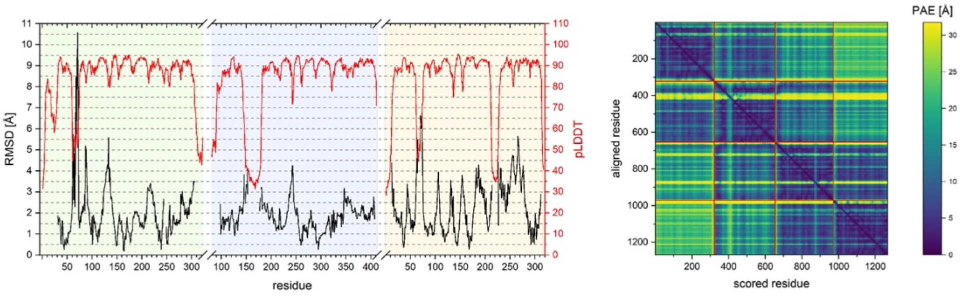

Supplementary Figure 3. Comparison of the different predicted, truncated septin structures with their ground truth structures extracted from the crystallized tetramer. The left panel shows alignments between the three-dimensional predicted models (magenta) and the crystal structure (light-grey). In parentheses AlphaFold-Multimer's model confidence is provided with 1.0 being the highest possible value. Alignments were performed using TM-align. The middle panel shows the RMSD-C_α for the aligned residues, plotted together with the pLDDT score for each residue following the color code of Figure 1. The right panel shows the plotted predicted aligned error (PAE), displaying the expected positional deviation in Å between residue x in the prediction compared to the ground truth structure when a local alignment on residue y is performed (using the C, N and $C\alpha$-atoms). The PAE is capped at 31.75 Å. The red lines in the PAE plots separate the different intra- and inter-subunit regions. The residue numbering starts with 1 corresponding to the first predicted residue in the most rod-internal subunit and increases traveling further to the termini of an octameric septin rod.

Supplementary Table 1. Evaluation of the AlphaFold predictions for the truncated septin constructs and the full-length $Cdc10_{NC}Cdc10$ dimer. The predictions with the highest model confidence were aligned to the different structures present in the crystallized septin tetramer. In parentheses AlphaFold-Multimer's model confidence is given. The resulting RMSD-Cα (bold letters) and $RMSD_{all\text{-}atom}$ values are listed. Since the α0 helix is inaccurately predicted when the respective NC binding partner is missing, we provide also RMSD values with the α0 helix excluded (values in italic). The comparison was performed between the predicted dimers/tetramer and the corresponding parts of the crystal structure.

**Aligned experimental structure**	**AlphaFold-Multimer constructs**				
	$Cdc10_{NC}Cdc10$ (0.873)	$Cdc10_{G}Cdc3$ (0.860)	$Cdc3_{NC}Cdc12$ (0.880)	$Cdc12_{G}Shs1$ (0.881)	Cdc11-rod (0.696)
Cdc10	**1.24 Å** | 1.89 Å	**1.34 Å** | 1.88 Å	-	-	**1.40 Å** | 1.90 Å
$Cdc10_{G}Cdc3$	-	**1.40 Å** | 3.87 Å<br>***1.22 Å*** | *1.75 Å*	-	-	**1.42 Å** | 1.87 Å
Cdc3	-	**1.37 Å** | 5.00 Å<br>***0.97 Å*** | *1.54 Å*	**1.03 Å** | 1.59 Å	-	**1.04 Å** | 1.58 Å
$Cdc3_{NC}Cdc12$	-	-	**1.18 Å** | 1.77 Å	-	**1.78 Å** | 2.21 Å
Cdc12	-	-	**1.03 Å** | 1.74 Å	**1.50 Å** | 5.67 Å<br>***1.08 Å*** | *1.83 Å*	**1.17 Å** | 1.84 Å
$Cdc12_{G}Shs1$	-	-	-	**1.27 Å** | 4.21 Å<br>***1.04 Å*** | *1.67 Å*	-
Shs1	-	-	-	**0.92 Å** | 1.46 Å	-
$Cdc10_{G}Cdc3_{NC}Cdc12$	-	-	-	-	**2.34 Å** | 2.69 Å

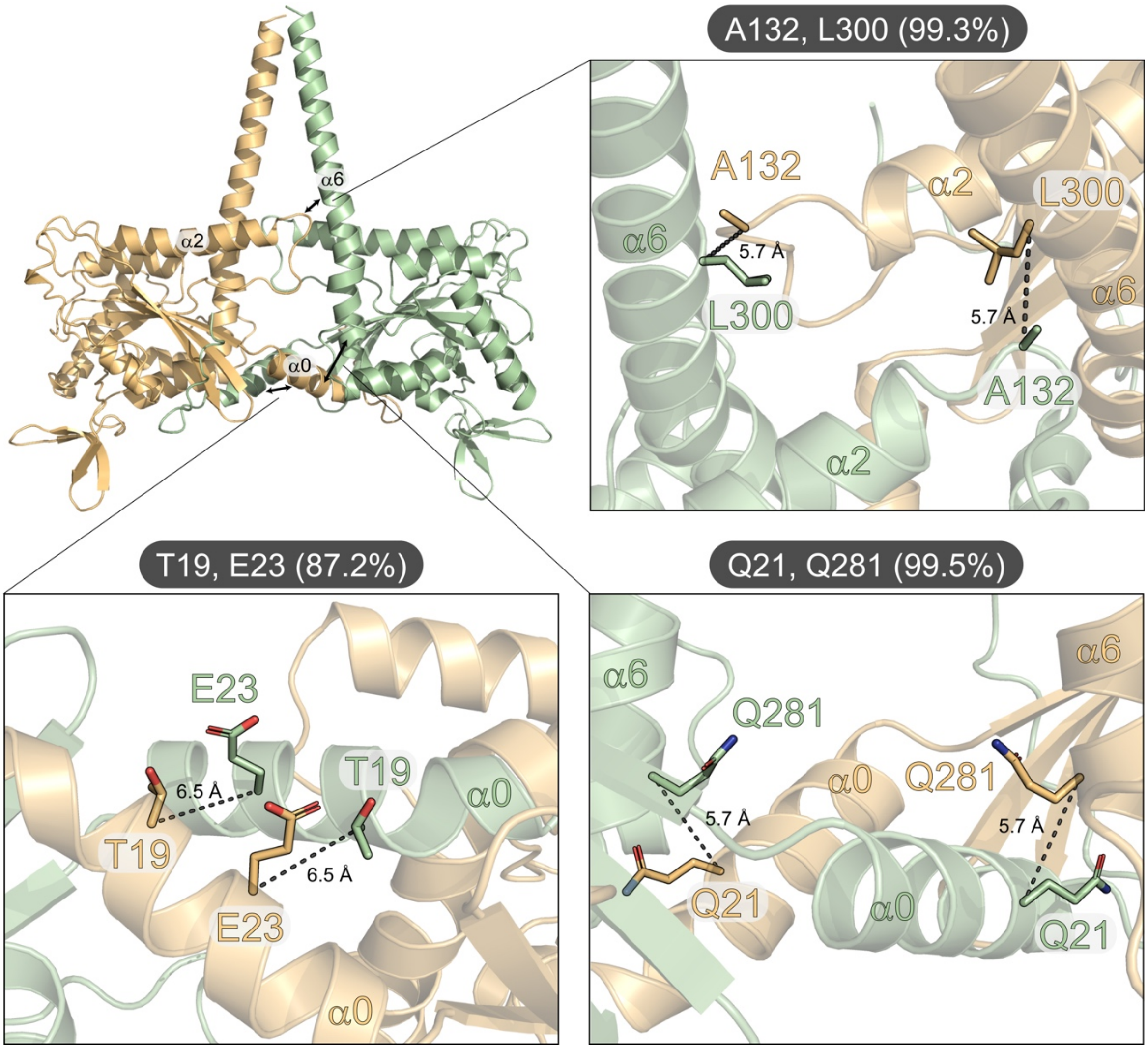

Supplementary Figure 4. Residues in the predicted Cdc10-Cdc10 interface selected as suitable for disulfide bridge engineering by the SSbondPre tool. The probability scores in percent are provided.

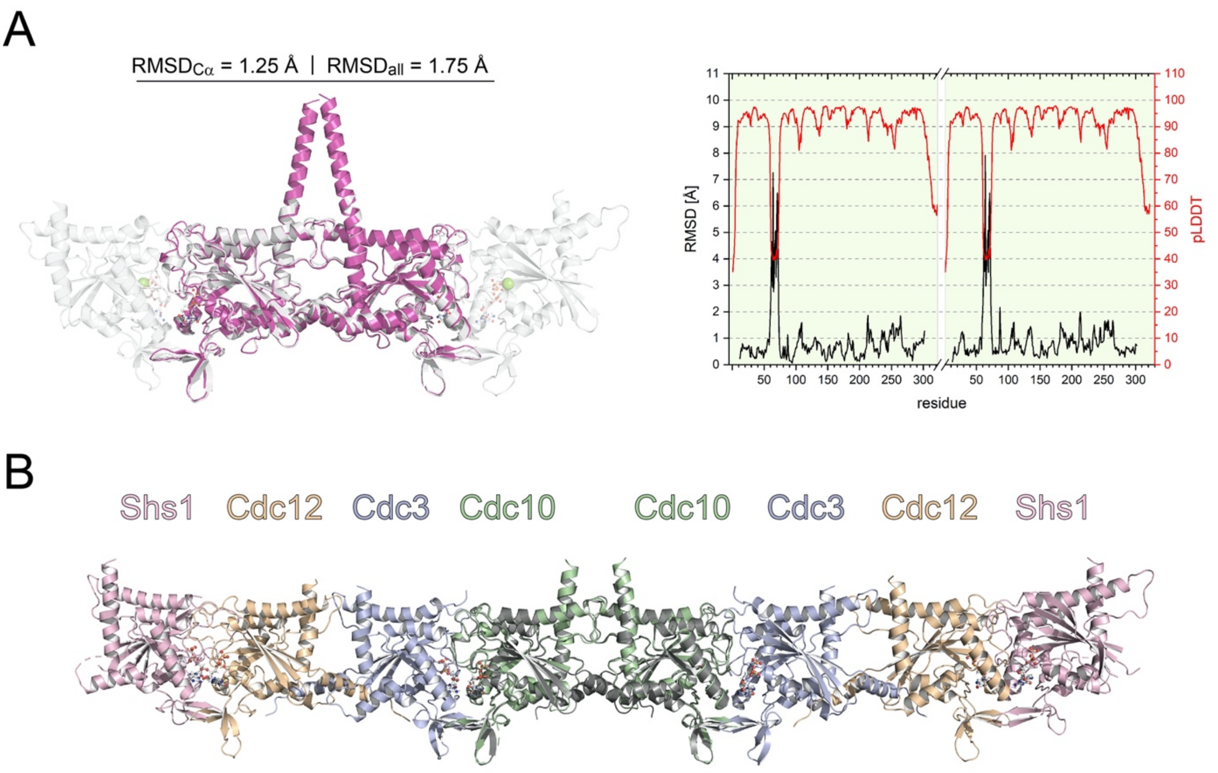

Supplementary Figure 5. The AlphaFold predicted Cdc10 dimer matches with the experimental structure. **A.** Comparison of the predicted Cdc10 dimer with the experimental Cdc10 interface from PDB-ID 8SGD. The left panel shows an alignment between the predicted model (magenta) and the crystal structure (light-grey). The right panel shows the RMSD-C_α for the aligned residues, plotted together with the pLDDT score for each residue following the color code of Figure 1. **B.** Structural model of an Shs1 containing octameric septin rod assembled from the tetrameric crystal structure and the experimental Cdc10 interface from 8SGD.

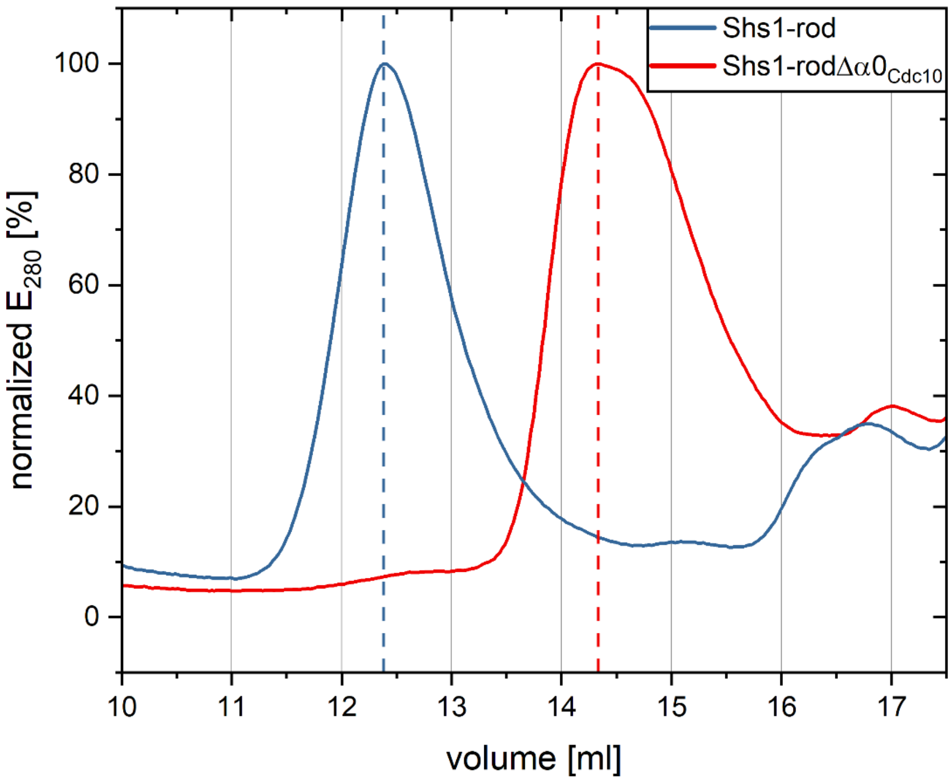

Supplementary Figure 6. Reference chromatograms for the full length and α0-helix truncated octameric septin rod. Shown are analytical size exclusion runs with normalized peak intensity.

A

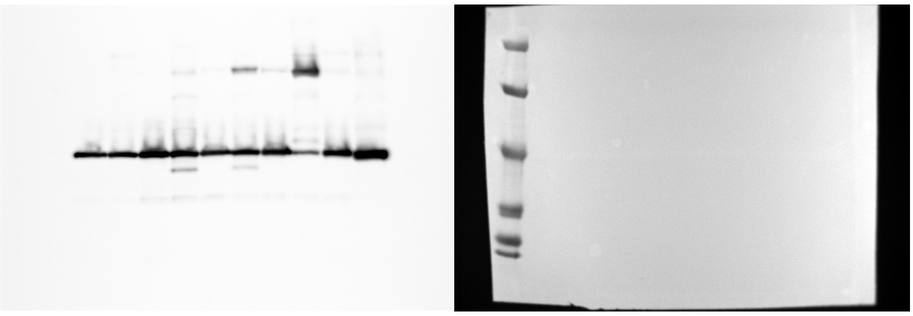

B

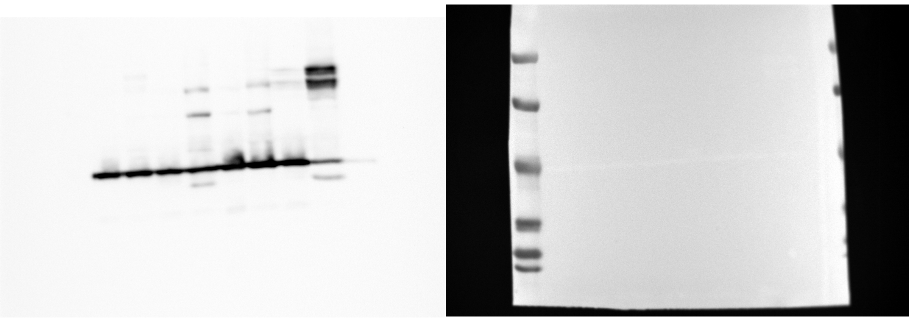

C

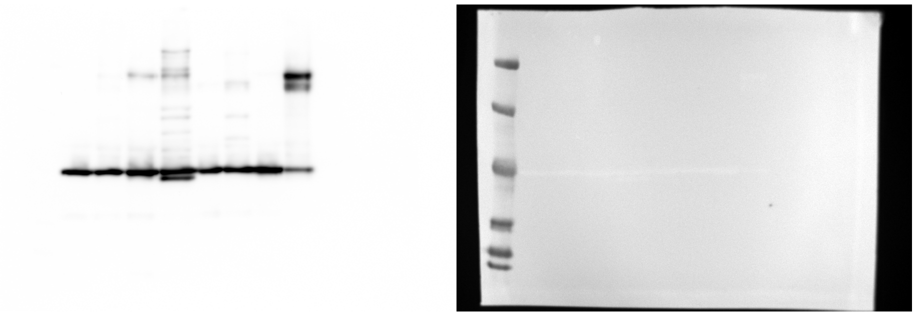

D

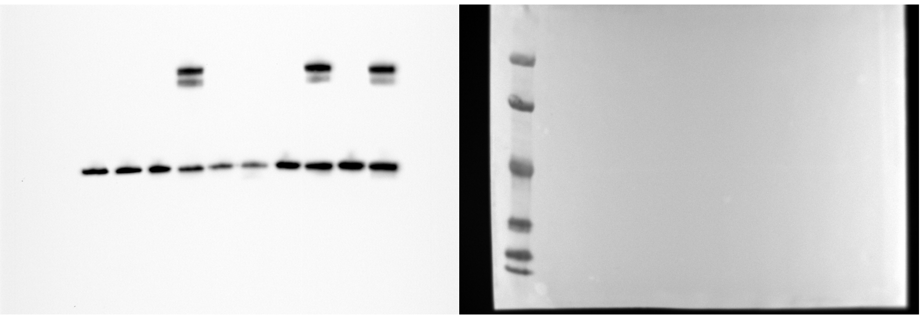

Supplementary Figure 7. Uncropped, unprocessed Western blots.

A-C. Western blots and corresponding markers for Figure 4B. A: left panel, B: middle panel, C: right panel.

D. Western blot and corresponding marker for Figure 7D.

Supplementary Methods

Plasmids and strains

Expression plasmids containing Cdc3, Cdc10, Shs1 and Cdc12 with truncated N and C-terminal extensions were created as described elsewhere[1].

Briefly, the ORFs of Cdc3 and Cdc10 were inserted into the bicistronic expression plasmid pACYCDuet-1 and those of Cdc12 and Shs11 into the compatible pETDuet-1 by standard cloning methods. To eliminate the His6 tag from pACYCDuet-1, we used Pci1, an isoschizomer of Nco1 which would cut in the ORF of Cdc3. C-terminal S-tag fusions were avoided by introducing stop codons. All generated plasmids are summarized in Table 1. Sequences of the oligonucleotide primers are available upon request from the authors.

The hydrophobic crest mutations V13A, F15A, I18A, I22A, L26A, L27A and F31A were introduced into the Cdc10 ORF by standard overlap-extension PCR using suitable primers yielding plasmids #6-#12.

Artificial disulfide bridges in the NC-interface were generated by introducing the mutations A132C, L300C, Q21C, Q281C, T19C, E23C and the respective double mutants into the Cdc10 ORF by standard overlap-extension PCR using suitable primers yielding plasmids #14-#25.

Expression constructs for human septins (#26-#29) were modifications of already designed constructs[2].

The ORF of Cdc10 and Cdc10 including the mutations V13A, F15A, I18A, I22A, L26A, L27A and F31A or lacking the α0 helix were cloned into the centromeric plasmid pRS313[3] containing a P_{MET17} promoter[4], yielding plasmids #33-#41.

The ORF of Cdc10 was cloned into the centromeric plasmid pRS316[3] (bearing an Ura3 prototrophy) and a P_{MET17} promoter[4], yielding plasmid #32. This rescue plasmid was transformed into the yeast strain JD47[5] by standard methods.

The wild type copy of *CDC10* in the resulting strain was replaced by a NAT cassette as described elsewhere[4] and plasmids #30 and #33-#41 were transformed into the resulting *CDC10* k.o. strain containing the rescue plasmid.

The strains were grown in YPD medium over-night and serial dilutions were subsequently spotted on suitable selection media containing FOA to drive out the rescue plasmid and on respective control media without FOA.

Supplementary Table 2 – Used and generated plasmids.

	Plasmid	Description	Origin
	pACYCDuet-1	2 lac-Operators: a) MCS1: His_6-Fusion peptide, b) MCS2: S-Fusion peptide; cat (Cm^R); lacI; P15A-Replicon	Novagen
	pETDuet-1	2 lac-Operators: a) MCS1: His_6-Fusion peptide, b) MCS2: S-Fusion peptide; bla (Amp^R); lacI; pBR322-Replicon	Novagen
1	pETDuet-1-Cdc12$_{M1\text{-}G314}$-Shs1$_{G21\text{-}S339}$	MCS1: His_6-Cdc12(G32–G314); MCS2: Shs1(G21–S339); bla (Amp^R);lacI; pBR322-Replicon	this work
2	pACYCDuet-1-Cdc3$_{Q81\text{-}A410}$-Cdc10$_{G30\text{-}R322}$	MCS1: Cdc3(Q81–A410); MCS2: Cdc10 (G30–R322); cat (Cm^R); lacI; p15A-Replicon	this work
3	pACYCDuet-1-Cdc3$_{Q81\text{-}A410}$-Cdc10$_{M1\text{-}R322}$	MCS1: Cdc3(Q81–A410); MCS2: Cdc10 (M1–R322); cat (Cm^R); lacI; lacI; p15A-Replicon	this work
4	pETDuet-1-Cdc12-Shs1	MCS1: His_6-Cdc12; MCS2: Shs1-Myc; bla (Amp^R);lacI; pBR322-Replicon	this work
5	pACYCDuet-1-Cdc3-Cdc10	MCS1: Cdc3; MCS2: Cdc10-S; cat (Cm^R); lacI; p15A-Replicon	this work
6	pACYCDuet-1-Cdc3-Cdc10(V13A)	MCS1: Cdc3; MCS2: Cdc10(V13A)-S; cat (Cm^R); lacI; p15A-Replicon	this work
7	pACYCDuet-1-Cdc3-Cdc10(F15A)	MCS1: Cdc3; MCS2: Cdc10(F15A)-S; cat (Cm^R); lacI; p15A-Replicon	this work
8	pACYCDuet-1-Cdc3-Cdc10(I18A)	MCS1: Cdc3; MCS2: Cdc10(I18A)-S; cat (Cm^R); lacI; p15A-Replicon	this work
9	pACYCDuet-1-Cdc3-Cdc10(I22A)	MCS1: Cdc3; MCS2: Cdc10(I22A)-S; cat (Cm^R); lacI; p15A-Replicon	this work
10	pACYCDuet-1-Cdc3-Cdc10(L26A)	MCS1: Cdc3; MCS2: Cdc10(L26A)-S; cat (Cm^R); lacI; p15A-Replicon	this work
11	pACYCDuet-1-Cdc3-Cdc10(L27A)	MCS1: Cdc3; MCS2: Cdc10(L27A)-S; cat (Cm^R); lacI; p15A-Replicon	this work
12	pACYCDuet-1-Cdc3-Cdc10(F31A)	MCS1: Cdc3; MCS2: Cdc10(F31A)-S; cat (Cm^R); lacI; p15A-Replicon	this work
13	pACYCDuet-1-Cdc3-Cdc10$_{G30\text{-}R322}$	MCS1: Cdc3; MCS2: Cdc10(G30-R322)-S; cat (Cm^R); lacI; p15A-Replicon	this work
14	pACYCDuet-1-Cdc3-Cdc10(A132C)	MCS1: Cdc3; MCS2: Cdc10(A132C)-S; cat (Cm^R); lacI; p15A-Replicon	this work
15	pACYCDuet-1-Cdc3-Cdc10(L300C)	MCS1: Cdc3; MCS2: Cdc10(L300C)-S; cat (Cm^R); lacI; p15A-Replicon	this work
16	pACYCDuet-1-Cdc3-Cdc10 (A132C, L300C)	MCS1: Cdc3; MCS2: Cdc10(A132C, L300C)-S; cat (Cm^R); lacI; p15A-Replicon	this work
17	pACYCDuet-1-Cdc3-Cdc10(Q21C)	MCS1: Cdc3; MCS2: Cdc10(Q21C)-S; cat (Cm^R); lacI; p15A-Replicon	this work
18	pACYCDuet-1-Cdc3-Cdc10(Q281C)	MCS1: Cdc3; MCS2: Cdc10(L26A)-S; cat (Cm^R); lacI; p15A-Replicon	this work
19	pACYCDuet-1-Cdc3-Cdc10 (Q21C, Q281C)	MCS1: Cdc3; MCS2: Cdc10(Q21C, Q281C)-S; cat (Cm^R); lacI; p15A-Replicon	this work
20	pACYCDuet-1-Cdc3-Cdc10(T19C)	MCS1: Cdc3; MCS2: Cdc10(T19C)-S; cat (Cm^R); lacI; p15A-Replicon	this work
21	pACYCDuet-1-Cdc3-Cdc10(E23C)	MCS1: Cdc3; MCS2: Cdc10(E23C)-S; cat (Cm^R); lacI; p15A-Replicon	this work

22	pACYCDuet-1-Cdc3-Cdc10 (T19C, E23C)	MCS1: Cdc3; MCS2: Cdc10(T19C, E23C)-S; cat (Cm^R); lacI; p15A-Replicon	this work
23	pACYCDuet-1-Cdc3-Cdc10 (I18A, A132C, L300C)	MCS1: Cdc3; MCS2: Cdc10(I18A, A132C, L300C)-S; cat (Cm^R); lacI; p15A-Replicon	this work
24	pACYCDuet-1-Cdc3-Cdc10 (L26A, A132C, L300C)	MCS1: Cdc3; MCS2: Cdc10(L26A, A132C, L300C)-S; cat (Cm^R); lacI; p15A-Replicon	this work
25	pACYCDuet-1-Cdc3-Cdc10 (L27A, A132C, L300C)	MCS1: Cdc3; MCS2: Cdc10(L27A, A132C, L300C)-S; cat (Cm^R); lacI; p15A-Replicon	this work
26	pETDuet-1-hSEPT6i1-hSEPT7i1	MCS1: His_6-hSEPT6i1; MCS2: hSEPT7i1; bla (Amp^R);lacI; pBR322-Replicon	Fischer et al. 2022
27	pACYCDuet-1-hSEPT2i1-hSEPT9i1$_{Q263\text{-}M586}$	MCS1: hSEPT2i1; MCS2: hSEPT9i1(Q263–M586); cat (Cm^R); lacI; p15A-Replicon	this work
28	pACYCDuet-1-hSEPT2i1-hSEPT9i1$_{Q263\text{-}M586}$(M288A)	MCS1: hSEPT2i1; MCS2: hSEPT9i1(Q263– M288A-M586); cat (Cm^R); lacI; p15A-Replicon	this work
29	pACYCDuet-1-hSEPT2i1-hSEPT9i1$_{Q263\text{-}M586}$(F297A)	MCS1: hSEPT2i1; MCS2: hSEPT9i1(Q263– F297A-M586); cat (Cm^R); lacI; p15A-Replicon	this work
30	pRS313	bla (Amp^R); HIS3; CEN6/ARSH4	Sikorski & Hieter 1987
31	pRS316	bla (Amp^R); URA3; CEN6/ARSH4	Sikorski & Hieter 1987
32	pRS316-Cdc10	MCS: P_{MET17}-Cdc10; bla (Amp^R); URA3; CEN6/ARSH4	this work
33	pRS313-Cdc10	MCS: P_{MET17}-Cdc10; bla (Amp^R); HIS3; CEN6/ARSH4	this work
34	pRS313-Cdc10(V13A)	MCS: P_{MET17}-Cdc10(V13A); bla (Amp^R); HIS3; CEN6/ARSH4	this work
35	pRS313-Cdc10(F15A)	MCS: P_{MET17}-Cdc10(F15A); bla (Amp^R); HIS3; CEN6/ARSH4	this work
36	pRS313-Cdc10(I18A)	MCS: P_{MET17}-Cdc10(I18A); bla (Amp^R); HIS3; CEN6/ARSH4	this work
37	pRS313-Cdc10(I22A)	MCS: P_{MET17}-Cdc10(I22A); bla (Amp^R); HIS3; CEN6/ARSH4	this work
38	pRS313-Cdc10(L26A)	MCS: P_{MET17}-Cdc10(L26A); bla (Amp^R); HIS3; CEN6/ARSH4	this work
39	pRS313-Cdc10(L27A)	MCS: P_{MET17}-Cdc10(L27A); bla (Amp^R); HIS3; CEN6/ARSH4	this work
40	pRS313-Cdc10(F31A)	MCS: P_{MET17}-Cdc10(F31A); bla (Amp^R); HIS3; CEN6/ARSH4	this work
41	pRS313-Cdc10$_{G30\text{-}R322}$	MCS: P_{MET17}-Cdc10(G30-R322); bla (Amp^R); HIS3; CEN6/ARSH4	this work

Supplementary Table 3 – Plasmid combinations for the various protein purifications

Protein purification purpose	Employed plasmids	Reference purification setup
Septin Shs1 tetramer for cystallization	#1 and #2	this work
Reconstitution of Shs1 octamer for analytical SEC	#1 and #3	Renz et al.
Full length Shs1 octamer for analytical SEC	#4 and #5	Renz et al.
Hydrophobic crest mutants and $\Delta\alpha0_{Cdc10}$, for analytical SEC	#4 and #6-#13	Renz et al.
Artificial disulfide bridges	#4 and #14-#25	this work (non-reducing IMAC)
Human septin octamer	#26 and #27-#29	Fischer et al. and this work

Mass spectrometry

5 µg of purified septin preparation were reduced with 5 mM DTT for 20 min at RT, alkylated with iodoacetamide for 20 min at 37 °C and diluted with 50 mM ammonium bicarbonate. Trypsin was added in a 1:50 enzyme-protein ratio and digested overnight at 37 °C. Peptides were cleaned up using self-made C18-STAGE tips (Empore C18 Extraction Disk, 3M) and following evaporation dissolved in 15 µl 0.1% TFA.

Samples were measured using an LTQ Orbitrap Velos Pro system (Thermo Fisher Scientific) online coupled to an U3000 RSLCnano (Thermo Fisher Scientific).

Database search was performed using MaxQuant Ver. 1.6.3.4 (www.maxquant.org)[6]. For peptide identification the build-in Andromeda search engine[7] was employed to correlate MS/MS spectra with the respective septin protein sequences obtained from www.uniprot.org.

Protein intensities were calculated as the sum of all identified peptide intensities (unique and razor peptides) belonging to the respective protein group.

Supplementary References

1. Renz, C., Johnsson, N. & Gronemeyer, T. An efficient protocol for the purification and labeling of entire yeast septin rods from E.coli for quantitative in vitro experimentation. *BMC Biotechnol.* **13**, 60 (2013).
2. Fischer, M., Frank, D., Rösler, R., Johnsson, N. & Gronemeyer, T. Biochemical Characterization of a Human Septin Octamer. *Front. cell Dev. Biol.* **10**, 771388 (2022).
3. Sikorski, R. S. & Hieter, P. A system of shuttle vectors and yeast host strains designed for efficient manipulation of DNA in Saccharomyces cerevisiae. *Genetics* **122**, 19–27 (1989).
4. Dünkler, A. *et al.* Type V myosin focuses the polarisome and shapes the tip of yeast cells. *J. Cell Biol.* **220**, (2021).
5. Dohmen, R. J. *et al.* An Essential Yeast Gene Encoding a Homolog of Ubiquitin-activating Enzyme. *J. Biol. Chem.* **270**, 18099–18109 (1995).
6. Cox, J. & Mann, M. MaxQuant enables high peptide identification rates, individualized p.p.b.-range mass accuracies and proteome-wide protein quantification. *Nat. Biotechnol.* **26**, 1367–72 (2008).
7. Cox, J. *et al.* Andromeda: a peptide search engine integrated into the MaxQuant environment. *J. Proteome Res.* **10**, 1794–805 (2011).
