# Supplementary material for: The structure of a tetrameric septin complex reveals a hydrophobic element essential for NC-interface integrity
Source: Commun Biol. 2024 Jan 6;7:48. doi: 10.1038/s42003-023-05734-w (PMC10771490; doi:10.1038/s42003-023-05734-w)
Supplement: Supplementary file 3 — Description of Additional Supplementary Files [file 42003_2023_5734_MOESM3_ESM.pdf]

Description of Additional Supplementary Files

File name: Supplementary Data 1
Description: All generated AlphaFold models in PDB format within one single zip folder.
